# Genome Sequencing of *Amomum tsao-ko* Provides Novel Insight Into Its Volatile Component Biosynthesis

**DOI:** 10.3389/fpls.2022.904178

**Published:** 2022-06-01

**Authors:** Fenghui Sun, Chaochao Yan, Yunyun Lv, Zhonghui Pu, Zedong Liao, Wei Guo, Min Dai

**Affiliations:** ^1^School of Laboratory Medicine, Chengdu Medical College, Chengdu, China; ^2^Chengdu Institute of Biology, Chinese Academy of Sciences, Chengdu, China; ^3^College of Life Science, Neijiang Normal University, Neijiang, China; ^4^Sichuan Provincial Engineering Laboratory for Prevention and Control Technology of Veterinary Drug Residue in Animal-Origin Food, Chengdu Medical College, Chengdu, China

**Keywords:** genome, gene family expansion, terpenoid biosynthesis, flavonoid metabolic process, *Amomum tsao-ko*

## Abstract

As an important economic and medicinal crop, *Amomum tsao-ko* is rich in volatile oils and widely used in food additives, essential oils, and traditional Chinese medicine. However, the lack of the genome remains a limiting factor for understanding its medicinal properties at the molecular level. Here, based on 288.72 Gb of PacBio long reads and 105.45 Gb of Illumina paired-end short reads, we assembled a draft genome for *A. tsao-ko* (2.70 Gb in size, contig N50 of 2.45 Mb). Approximately 90.07% of the predicted genes were annotated in public databases. Based on comparative genomic analysis, genes involved in secondary metabolite biosynthesis, flavonoid metabolism, and terpenoid biosynthesis showed significant expansion. Notably, the *DXS*, *GGPPS*, and *CYP450* genes, which participate in rate-limiting steps for terpenoid backbone biosynthesis and modification, may form the genetic basis for essential oil formation in *A. tsao-ko*. The assembled *A. tsao-ko* draft genome provides a valuable genetic resource for understanding the unique features of this plant and for further evolutionary and agronomic studies of Zingiberaceae species.

## Introduction

*Amomum tsao-ko* (Zingiberaceae) is a perennial herbaceous plant widely distributed in southwest China and Vietnam. As a traditional Chinese medicine, the dried fruits of *A. tsao-ko* are used to treat malaria, throat infections, abdominal pain, dyspepsia, nausea, stomach disorders, vomiting, and diarrhea (Tang et al., 2010). Clinical and animal trials indicate that *A. tsao-ko* exhibits a wide range of pharmacological activities, including antioxidant, cytotoxic, and antimicrobial activities (Li et al., 2014; Hong et al., 2015; Dai et al., 2016; Lin et al., 2021). The essential oil of *A. tsao-ko* and its polyphenol extract can modulate gut microbiota and alleviate hypercholesterolemia (Liu et al., 2021). The ethanol extract of *A. tsao-ko* can improve dyslipidemia-related indices, including plasma levels of total cholesterol, low-density lipoprotein, high-density lipoprotein, and atherogenesis, in mice on high-carbohydrate diets (Park et al., 2021). It is also a common food additive and spice, which can develop food flavor while retaining medicinal effects.

*Amomum tsao-ko*-based essential oils include terpenoids, diarylheptanoids, bicyclic nonanes, and phenols, which may account for the plant’s medicinal properties (Hong et al., 2015; Cui et al., 2017; Sim et al., 2019). The monoterpene alcohol geraniol is a widely used fragrance ingredient and one of the main components (13.69%) of *A. tsao-ko* essential oil (Lapczynski et al., 2008). Geraniol shows significant inhibitory effects against *Staphylococcus aureus*, a pathogen responsible for many infections (Long et al., 2020, 2022). Geraniol can also improve endothelial function in high-fat diet-fed mice by inhibiting oxidative stress (Wang et al., 2016). Eucalyptol (1,8-cineole), another component of *A. tsao-ko* essential oil, displays antioxidant, antibacterial, anti-inflammatory, and insecticidal activities (Cai et al., 2021). Furthermore, *A tsao-ko* extract flavonoids show excellent antioxidative and antidiabetic activity (Zhang et al., 2022). While these studies have revealed the main medicinal properties and constituents of *A. tsao-ko*, the lack of a genome limits our understanding of the genomic and molecular basis of its volatile component biosynthesis.

Herein, we generated a draft genome assembly of *A. tsao-ko* using PacBio long reads and Illumina paired-end short reads. We constructed a genome-wide phylogeny of *A. tsao-ko* with eight other available plant genomes. Comparative genomic analysis indicated that gene families involved in the synthesis of terpenoids were expanded, which may provide clues for exploring the biosynthesis of volatile components in *A. tsao-ko*. Overall, this draft genome provides a valuable genetic resource for in-depth biological and evolutionary studies and for genetic improvement of *A. tsao-ko*.

## Materials and Methods

### Sample Collection, Sequencing, and Data Qualification

**FIGURE 1:**
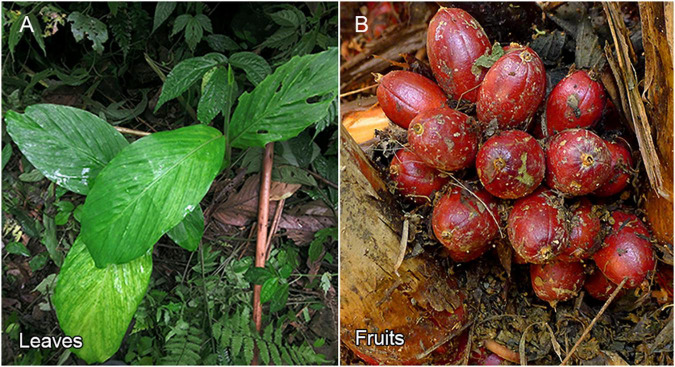
Morphological features of *A. tsao-ko.* **(A)** The leaves of *A. tsao-ko*. **(B)** The fruits of *A. tsao-ko*.

We collected fresh leaves from an adult *A. tsao-ko* plant (Figure 1) growing in Guangxi Zhuang Autonomous Region, southern China. Total genomic DNA was extracted, and DNA quantification and quality testing were determined using NanoDrop 2000 spectrophotometry (Thermo Fisher Scientific), gel electrophoresis, and Qubit fluorometry (Invitrogen). For short-read sequencing, paired-end libraries with a 350-bp insert size were prepared following the manufacturer’s instructions and then sequenced on the Illumina NovaSeq 6000 platform. Clean reads were obtained by removing contaminated reads from low-quality data. The PacBio single-molecule real-time (SMRT) bell library was constructed using a SMRTbell^®^ Express Template Prep Kit 2.0 (Pacific Biosciences, PN 101-853-100). The library was prepared for sequencing on the PacBio Sequel II system (Pacific Biosciences, CA, United States). After adapter removal, we obtained subreads. A total of 105.45 Gb of raw paired-end short reads and 288.72 Gb of PacBio subreads were generated, which were reduced by 0.09 and 0.12%, respectively, after trimming and quality control (Supplementary Table 1). Average subread length in the two PacBio libraries was 22,362 and 22,751 bp, with a mean insert length of 23,106 and 23,457 bp, respectively (Supplementary Table 2).

Fresh leaves were also prepared for RNA sequencing to aid in genome annotation. Total RNA was extracted using a QIAGEN^®^ RNA Mini Kit following the manufacturer’s protocols. RNA purity and integrity were assessed using the NanoPhotometer^®^ spectrophotometer (IMPLEN, CA, United States) and RNA Nano 6000 Assay Kit and Bioanalyzer 2100 system (Agilent Technologies, CA, United States), respectively. The RNA sequencing library was constructed using a NEBNext^®^ Ultra™ RNA Library Prep Kit for Illumina^®^ (NEB, United States) following the manufacturer’s instructions. RNA sequencing was performed on the Illumina NovaSeq 6000 platform. Low-quality reads were excluded using Trimmomatic v.0.36.23 (Bolger et al., 2014). After quality control, 7.42 Gb of clean data retained for genome annotation (Supplementary Table 1).

### Genome Assembly and Quality Assessment

The 1C value of *A. tsao-ko* was measured using flow cytometry (Cytoflex, Bio-Rad, United States) with propidium iodide (PI) as the DNA stain and *Vigna radiata* as reference standard plant. The genome size of *V. radiate* is 579 Mb as described previously (Arumuganathan and Earle, 1991). Three biological repeats were performed and the mixture of two plants as internal standard. We then assembled the genome with PacBio long reads using mecat2 (Xiao et al., 2017) and polished the assembly with PacBio long reads and short pair-end reads using NextPolish v1.4.0.^1^ The assembly quality was assessed using the Embryophyta gene sets in BUSCO v3.1.0 with genome mode and kmer-spectra analysis, referring to the previous studies (Simao et al., 2015; Mapleson et al., 2017; Yang et al., 2019; Yang F. S. et al., 2020; Wang et al., 2021). We also applied LTR assembly index (LAI) to evaluate the continuity of the assembly based on the ratio of whole LTR retrotransposons (LTR-RTs) (Ou et al., 2018). The genome quality is at a draft level when 0 < LAI ≤ 10, at reference level when 10 < LAI ≤ 20 and at gold level when 20 ≤ LAI (Ou et al., 2018).

### Genome Annotation and Analysis of Transposons

MITE-Hunter v1.0 was used to annotate the miniature inverted repeat transposable elements (MITEs) with default parameters (Han and Wessler, 2010). The LTR-retriever v2.82 pipeline, which combined results from LTRharvest (with parameter: -similar 90 -vic 10 -seed 20 -seqids yes -minlenltr 100 -maxlenltr 7000 -mintsd 4 -maxtsd 6 -motif TGCA –motifmis 1) and LTR-Finder v1.07 (-D 15000 -d 1000 -L 7000 -l 100 -p 20 -C -M 0.9), was used to identify long terminal repeat retrotransposons (LTR-RT) (Xu and Wang, 2007; Ellinghaus et al., 2008; Ou and Jiang, 2018). We then searched repetitive sequences in Repbase v20170127 using RepeatMasker v4.1.0 (Tarailo-Graovac and Chen, 2009) and constructed the repetitive sequence library by combining results from the MITE-Hunter and LTR-retriever pipelines. After masking the genome, *de novo* repeat annotation of *A. tsao-ko* was performed using RepeatModeler v2.0.1 (Flynn et al., 2020). Non-coding RNA was annotated against the sequence in the Rfam database and transfer RNA (tRNA) was predicted using tRNAscan-SE 2.0^2^ (Chan et al., 2021).

A hybrid strategy of *de novo* gene prediction, homology-based prediction, and transcriptome alignment was applied for gene structural prediction using GeMoMa-1.6.1, Augustus v3.0.3, SNAP v6.0,^3^ GlimmerHMM v3.0.4, and GeneMark-ET v4.57 (Stanke and Waack, 2003; Majoros et al., 2004; Hoff et al., 2016; Keilwagen et al., 2016). The predicted results were combined using EVM^4^ and the untranslated region (UTR) and alternative splicing were predicted using PASA v2.0.1 (Haas et al., 2008). To determine the functional annotation of gene models, Diamond v0.9.31 (Buchfink et al., 2014) analysis with default parameters was performed against protein databases, including NR (non-redundant protein sequences in NCBI), SwissProt, and eggNOG, Gene Ontology (GO), describing molecular function (MF), cellular component (CC), and biology process (BP) terms, was annotated using Blast2GO (Conesa and Götz, 2008). Kyoto Encyclopedia of Genes and Genomes (KEGG) analysis was used to annotate the pathway genes involved. The motifs and domains of each gene model were predicted using InterProScan v5.18-57.0 (Jones et al., 2014).

### Phylogenetic Analyses

Referring to the genome study of a Zingiberaceae plant (Chakraborty et al., 2021), the orthologous groups among nine plant species, including *Ananas comosus* (GCF_001540865.1), *Asparagus officinalis* (GCA_001876935.1), *Arabidopsis thaliana* (GCF_000001735.4), *Amborella trichopoda* (GCF_000471905.2), *Musa acuminate* (GCF_000313855.2), *Musa balbisiana* (GCA_004837865.1), *Oryza sativa* (GCF_001433935.1), *Sorghum bicolor* (GCF_000003195.3), and *A. tsao-ko*, were constructed using OrthoFinder v2.2.7 (Emms and Kelly, 2019). Single-copy genes from the nine species were extracted and the proteins for each gene were aligned. All alignments were combined to a supergene for each species to construct a phylogenetic tree using RAxML v8.2.12 (Stamatakis, 2014). Divergence time was estimated under the relaxed clock model using MCMCTree in PAML v4.9 (Yang, 2007). Three calibration points (the ancestors of *Asp. officinalis* and *M. acuminate*; *Ara. thaliana* and *Amb. trichopoda*; *O. sativa* and *S. bicolor*) for the divergence analysis were obtained from the TimeTree database (Kumar et al., 2017).

### Analysis of Gene Family Expansion and Contraction

The results obtained from OrthoFinder v2.2.7 were used for gene family analysis. Genes that were unassigned (could not be clustered into any gene family) or found in only one species were considered species-specific. Gene family expansion and contraction analysis was performed using CAFE v4.2.1. A family-wide Viterbi *P*-value < 0.05 was defined as a significantly expanded or contracted gene family. Visualization used performed using python scripts.^5^

### Functional and Pathway Enrichment Analysis

Enrichment analysis was performed to provide insights into the biological functions of species-specific genes and expanded genes families. GO and KEGG analyses were performed using the R package clusterProfiler v4.0 (Wu et al., 2021). The *A. tsao-ko* annotated results were set as background genes. Enriched terms with a corrected *P*-value < 0.05 were considered significantly over-represented.

## Results and Discussion

### *De novo* Assembly of *Amomum tsao-ko*

We estimate the genome size of *A. tsao-ko* with flow cytometry using *Vigna radiata* as reference standard and the results showed that the genome size of *A. tsao-ko* was approximately 3.17 Gb (Supplementary Figure 1). We used PacBio long reads to construct the primary assembly and used long reads and Illumina paired-end short reads to polish the assembly. The final *A. tsao-ko* assembly size was 2.70 Gb, with a contig N50 of 2.45 Mb. In comparison to other Zingiberaceae genomes, the *A. tsao-ko* genome had a higher contig N50 than turmeric (contig N50 = 0.1 Mb), but a lower contig N50 than ginger (contig N50: 4.68 Mb for haplotype 1 and 5.28 Mb for haplotype 0) (Chakraborty et al., 2021; Li et al., 2021). Average GC content in the *A. tsao-ko* genome was 41.07%, higher than that of ginger (39.20%) and turmeric (38.75%). We evaluated assembly quality using BUSCO, resulting in 1,565 (97.0%) complete BUSCOs, including 1,117 (69.2%) single-copy BUSCOs, and 448 (27.8%) duplicated BUSCOs.

**FIGURE 2:**
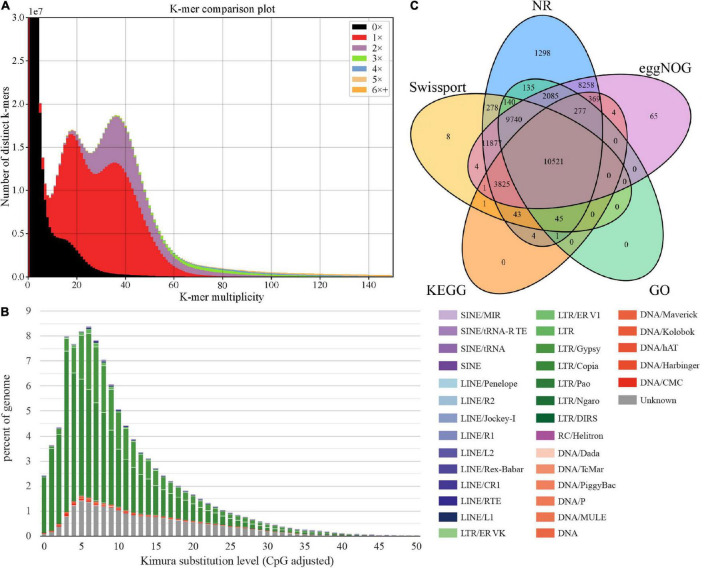
Genome size assessment and function annotation. **(A)** *K*-mer spectra between WGS (whole-genome sequencing) and the assembly. **(B)** TE expansion patterns of *A. tsao-ko*. **(C)** Venn diagram for functional annotation of genes in different databases.

The *k*-mer Analysis Toolkit (KAT) can be used to assess errors, bias, and genome quality (Mapleson et al., 2017). We used KAT to estimate the assembly quality through pairwise comparison of *k*-mers present in both the input reads and assembly. As shown in Figure 2A, reads in black represent absence in the assembly, including incorrect and low-depth *k*-mers, accounting for a relatively small proportion. These suggests the current assembly covered most short reads *k*-mers content, with relatively high completeness (evaluation score 96.83%). We also observed multimodal spectra in the assembly, which may be influenced by heterozygous contents or by tetrapods of *A. tsao*-ko (Parthasarathy and Prasath, 2012). A previous study of 100 Archea, Bacteria, and Eukaryota species based on *k*-mer spectra indicates that species with multimodal spectra are consistent with tetrapods (Chor et al., 2009). Thus, KAT analysis of *A. tsao-ko* assembly indicated that the genome is complex and cannot be explained by a simple probabilistic model, such as genome size estimation based on Poisson distribution of *k*-mer depth. GC-depth distribution showed two peaks at ∼20× and ∼40×, also suggesting the complex of *A. tsao-ko* genome (high heterozygosity or polyploidy) (Supplementary Figure 2).

Containing appropriately 1,500 species, Zingiberaceae is one of the largest monocotyledonous families, producing valuable medicinal materials and spices. At present, however, only a few Zingiberaceae genomes have been reported, e.g., *Zingiber officinale* and *Curcuma longa* (Chakraborty et al., 2021; Li et al., 2021). Furthermore, within the *Amomum* genus, only a limited number of chloroplast genomes have been described (Zhang et al., 2019; Yang L. et al., 2020). The lack of whole-genome data has severely impeded our understanding of essential oil biosynthesis in *A. tsao-ko*. Thus, the genome reported in this study should serve as an important resource for further genetic improvement and for exploring the molecular basis of essential oil biosynthesis.

### Repeat Identification and Gene Model Prediction

We annotated the repetitive sequences based on the *de novo* repeat sequence database of *A. tsao-ko* combined with Repbase v20170127. Results showed that 89.15% (2.41 Gb) of the *A. tsao-ko* genome contained repetitive sequences (Supplementary Table 3), much higher than that reported for other Zingiberaceae plants (e.g., ∼70% for turmeric and ∼57% for ginger) (Chakraborty et al., 2021; Li et al., 2021). Similar to turmeric [27.37% long terminal repeats (LTRs)], the LTR retrotransposons in the *A. tsao-ko* genome were also the most abundant transposable elements, namely LTR_*Copia* and LTR_*Gypsy* (54.71%, Figure 2B and Supplementary Table 3). Simple tandem repeats accounted for a relatively low proportion (0.7%) of the *A. tsao-ko* genome. In addition, the LAI of the assembly was estimated as 17.85, suggesting a relatively high completeness.

In total, 54,379 protein-coding genes were annotated in the *A. tsao-ko* genome using the three strategies described above, and the number of genes predicted in *A. tsao-ko* (54,379), ginger (∼39,000), and turmeric (50,401) varied. Mean gene length was 5,613 bp and number of transcripts was 57,658, with 5.3 exons per transcript (Supplementary Table 4). Approximately 90.07% of the predicted genes were annotated in five public databases, including NR (89.92%), eggNOG (86.48%), SwissProt (67.09%), KEGG (27.75%), and GO (42.19%). The Venn diagram in Figure 2C shows that 10,521 (19.35%) protein-coding genes were simultaneously annotated in the five databases. GO annotation classified these genes into three main categories, i.e., biological process (e.g., oxidation-reduction process, proteolysis, and protein phosphorylation), cellular component (e.g., integral component of membrane, membrane, and nucleus), and molecular function (e.g., adenosine triphosphate (ATP) binding, metal ion binding, and zinc ion binding) (Supplementary Figure 3). In addition, non-coding RNAs were identified, resulting in 3,335 ribosomal RNAs (rRNAs), 234 microRNAs (miRNAs), and 1,417 tRNAs (Supplementary Table 5). Collinearity analysis found that the collinear duplicates occupied about 48% of all genes in *A. tsao-ko* genome. The Ks distribution displays a clear Ks peak (Supplementary Figure 4) at about 0.35 that should be resulted from a whole-genome duplication event, which leads to a tetraploid generation.

### Single-Copy Orthologous Phylogeny

**FIGURE 3:**
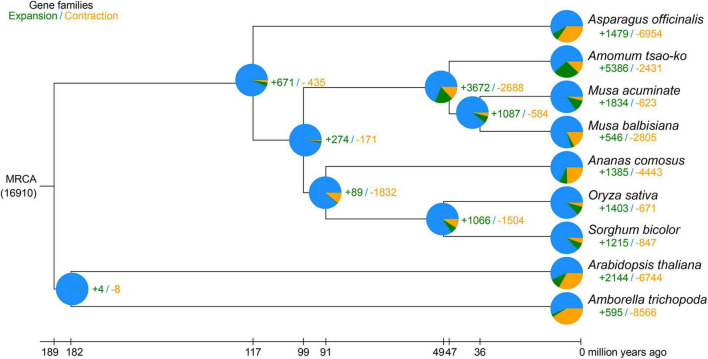
Phylogeny of *A. tsao-ko* and gene family analysis. The numbers in green represent the expanded gene families, the numbers in yellow represent the contracted gene families. MRCA, most recent common ancestor.

Nine plant species, i.e., *A. tsao-ko*, *Ana. comosus*, *Asp. officinalis*, *Ara. thaliana*, *Amb trichopoda*, *M. acuminate*, *M. balbisiana*, *O. sativa*, and *S. bicolor*, were selected for orthologous group identification (Supplementary Table 6). In total, 1,288 single-copy orthologs shared among the species were extracted. We constructed a phylogenetic tree based on the 1,288 single-copy orthologs using RAxML, with *Ara. thaliana* and *Amb. trichopoda* set as the outgroups (Figure 3). The genome-wide phylogenetic positions of *A. tsao-ko* and selected species were supported by TIMETREE. Results showed that *M. acuminate*, *M. balbisiana*, and *A. tsao-ko* belonged to Zingiberales, and shared the same phylogenetic clade. Furthermore, *A. tsao-ko* separated from Musaceae approximately 30∼63 million years ago (MYA) and *Asp. officinalis*, as a member of Asparagales, showed early divergence among the monocotyledons.

### Analysis of Gene Families and Genes Involved in Flavonoid Metabolism

**FIGURE 4:**
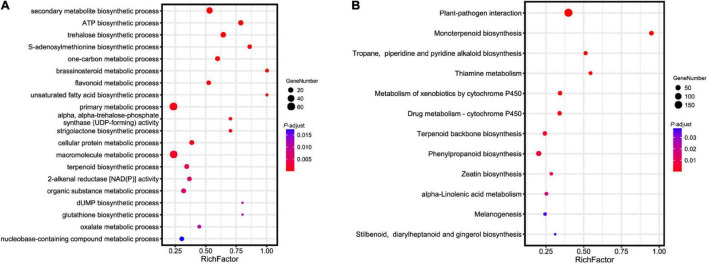
Functional and pathway enrichment analysis of expanded genes in *A. tsao-ko*. **(A)** GO enrichment analysis of expanded genes in *A. tsao-ko*. **(B)** KEGG enrichment analysis of expanded genes in *A. tsao-ko*.

To investigate the genetic basis of essential oil biosynthesis, we performed gene family expansion and contraction analysis of *A. tsao-ko* in comparison to the other eight species selected. Notably, 5,386 gene families showed significant expansion and 2,431 gene families showed significant contraction in *A. tsao-ko* (family-wide Viterbi *P*-value < 0.05, Figure 3). The expanded gene families were subjected to functional enrichment analysis (*P*-adjust cutoff of 0.05). The top 10 most significantly enriched terms included cellular macromolecule metabolic process (GO:0044260), endonuclease activity (GO:0004519), and peptidase activity (GO:0008233) (Supplementary Table 7). In addition, multiple biosynthetic and metabolic process-related terms were significantly enriched, including secondary metabolite biosynthetic process (GO:0044550), S-adenosylmethionine biosynthetic process (GO:0006556), one-carbon metabolic process (GO:0006730), and flavonoid metabolic process (GO:0009812) (Figure 4A). The essential oil of *A tsao*-*ko* is a secondary metabolite with strong biological activity and medicinal value and plays an important role in plant defense against disease, insects, and competition (Qin et al., 2021). Thus, the significant expansion of genes associated with secondary metabolism suggests enhancement of related functions.

Previous studies have suggested that *A. tsao-ko* shows potential as a novel drug for the treatment of type 2 diabetes due to the excellent antioxidative and antidiabetic activity of its flavonoids (Fang et al., 2019; Zhang et al., 2022). Here, flavonoid metabolic processes were significantly enriched by the *A tsao-ko* expanded genes, including *UGT71K1*, *RGGA*, and *CZOG2*. Among these genes, *UGT71K1* encodes a protein with chalcone and flavonol 2′-O-glycosyltransferase activity, as well as, glycosyltransferase activity toward quercetin isoliquiritigenin and butein (Gosch et al., 2010). Flavanols, as major components of flavonoids in *A. tsao-ko* extract, show antidiabetic potency (Fang et al., 2019; He et al., 2020, 2021). In addition, UGT71K1 can convert phloretin to phlorizin, a potent antioxidant with antidiabetic effects that competitively inhibits sodium-glucose symporters (Gosch et al., 2010).

### Pathway Enrichment Analysis and Genetic Basis of Terpenoid Biosynthesis

We performed KEGG pathway enrichment analysis of the expanded genes. Results showed that the expanded genes in *A. tsao-ko* were significantly enriched in biosynthesis- [i.e., monoterpenoid biosynthesis (ko00902), terpenoid backbone biosynthesis (ko00900), tropane, piperidine and pyridine alkaloid biosynthesis (ko00960), and stilbenoid, diarylheptanoid and gingerol biosynthesis (ko00945)], metabolism- [e.g., metabolism of xenobiotics by cytochrome P450 (ko00980) and drug metabolism-cytochrome P450 (ko00982)], and immune-related pathways [e.g., plant-pathogen interaction (ko04626) and melanogenesis (ko04916)] (Figure 4B and Supplementary Table 8).

**FIGURE 5:**
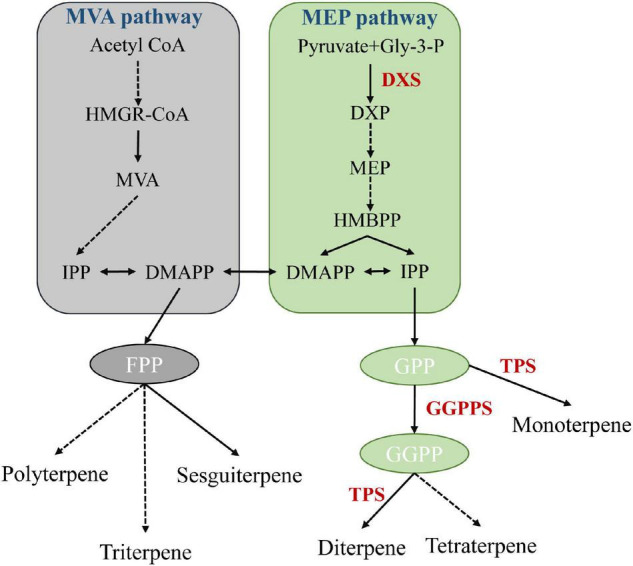
Schematic diagram of MEP and MVA pathways (Zhang et al., 2021). Each solid arrow represents a biosynthetic reaction step, and dashed arrows represent multiple-step reactions. HMGR: 3-hydroxy-3-methylglutary-CoA reductase, MVA: mevalonate, DMAPP: dimethylallyl pyrophosphate, IPP: isopentenyl pyrophosphate, DXP: 1-deoxy-D-xylulose-5-phosphate, DXR: 1-deoxy-D-xylulose-5-phosphate reductoisomerase, MEP: 2C-methyl-D-erythritol 4-phosphate pathway, GGPP: geranylgeranyl pyrophosphate.

Terpenoids, such as geraniol and eucalyptol, are the main components of *A. tsao-ko* essential oil, and show antioxidant, antidiabetic, antibacterial, anti-inflammatory, and insecticidal activities (Lapczynski et al., 2008; Dai et al., 2016; Wang et al., 2016; Cai et al., 2021). Biosynthesis of terpenoids in plants is a complex process, involving backbone biosynthesis and terpenoid synthesis and modification. In nature, mevalonate (MVA) and 2C-methyl-d-erythritol 4-phosphate (MEP), located in the cytoplasm and plastids, respectively, are two major pathways of terpenoid biosynthesis (Figure 5; Lei et al., 2021). We found that 1-deoxy-D-xylulose-5-phosphate synthase (including *DXS*, *DXS1*, and *DXS2*) and geranylgeranyl diphosphate synthase (*GGPPS*) genes were significantly expanded and enriched in the terpenoid backbone biosynthesis pathway. DXS can catalyze the condensation of pyruvate and d-glyceraldehyde 3-phosphate (GAP) to produce 1-deoxy-D-xylulose 5-phosphate (DXP), the first rate-limiting reaction of the MEP pathway (Hahn et al., 2001; Battistini et al., 2016). GGPP serves as a key precursor substrate of volatile and non-volatile terpenoids (Beck et al., 2013). *GGPPS* encodes an important enzyme involved in the synthesis of volatile and non-volatile terpenoids, constituting a key node that regulates carbon flow in the isoprenoid pathway (Zhang et al., 2021). Furthermore, based on Café pipeline analysis, terpene synthase (TPS) genes, including *TPS2*, *TPS4*, and *TPS10*, were expanded in *A. tsao-ko*, and significantly over-represented in the monoterpenoid biosynthesis pathway. TPSs can harness specific prenyl precursors to produce hemiterpenoids, monoterpenoids, sesquiterpenoids, diterpenoids, triterpenoids, and tetraterpenoids (Lei et al., 2021). Cytochrome P450 (CYP450) enzymes play critical roles in terpenoid skeleton modification and structural diversity (Zheng et al., 2019), and we found that related pathways were also over-represented in various expanded genes, such as *GSTU6*, *GSTUF*, and *GSTF1*. Overall, the expansion of genes encoding key rate-limiting enzymes in terpenoid synthesis and modification-related pathways may facilitate the synthesis of terpenoids, highlighting the biological activity and medicinal properties of *A. tsao-ko*.

## Conclusion and Future Perspectives

In this study, we assembled a draft genome of *A. tsao-ko*, which should provide valuable insights into the evolutionary history of Zingiberaceae. We further identified candidate genes involved in the biosynthesis of terpenoids, flavonoids, and other secondary metabolites, including several genes encoding key rate-limiting enzymes of the biosynthetic pathway. These results provide a genetic basis for the formation of main terpenoids and other secondary metabolites of *A. tsao-ko*, which is of great advantage for the manipulation of related enzymes and improvement of breeding of this important medicinal plant. However, given the complexity of the *A. tsao-ko* genome, further studies are needed.

## Data Availability Statement

The data presented in the study are deposited in the CNGB Sequence Archive (CNSA) of China National GeneBank DataBase (CNGBdb), accession number CNP0002802.

## Author Contributions

WG and MD conceived the study and worked on the approval of the manuscript. FS, WG, and MD prepared the initial manuscript draft. FS and ZL performed data analyses. CY finished evolutionary analysis. YL assembled the genome. ZP collected experimental samples and modified the manuscript. All authors contributed to the article and approved the submitted version.

## Conflict of Interest

The authors declare that the research was conducted in the absence of any commercial or financial relationships that could be construed as a potential conflict of interest.

## Publisher’s Note

All claims expressed in this article are solely those of the authors and do not necessarily represent those of their affiliated organizations, or those of the publisher, the editors and the reviewers. Any product that may be evaluated in this article, or claim that may be made by its manufacturer, is not guaranteed or endorsed by the publisher.

## Footnotes

1
https://github.com/Nextomics/NextPolish

2
http://lowelab.ucsc.edu/tRNAscan-SE/

3
http://snap.stanford.edu/snap/download.html

4
https://sourceforge.net/projects/evidencemodeler/

5
https://github.com/hahnlab/cafe_tutorial.git
