# Supplementary material for: Genome Sequencing of Amomum tsao-ko Provides Novel Insight Into Its Volatile Component Biosynthesis
Source: Front Plant Sci. 2022 Jun 1;13:904178. doi: 10.3389/fpls.2022.904178 (PMC9198571; doi:10.3389/fpls.2022.904178)
Supplement: Supplementary file 1 [file Data_Sheet_1.docx]

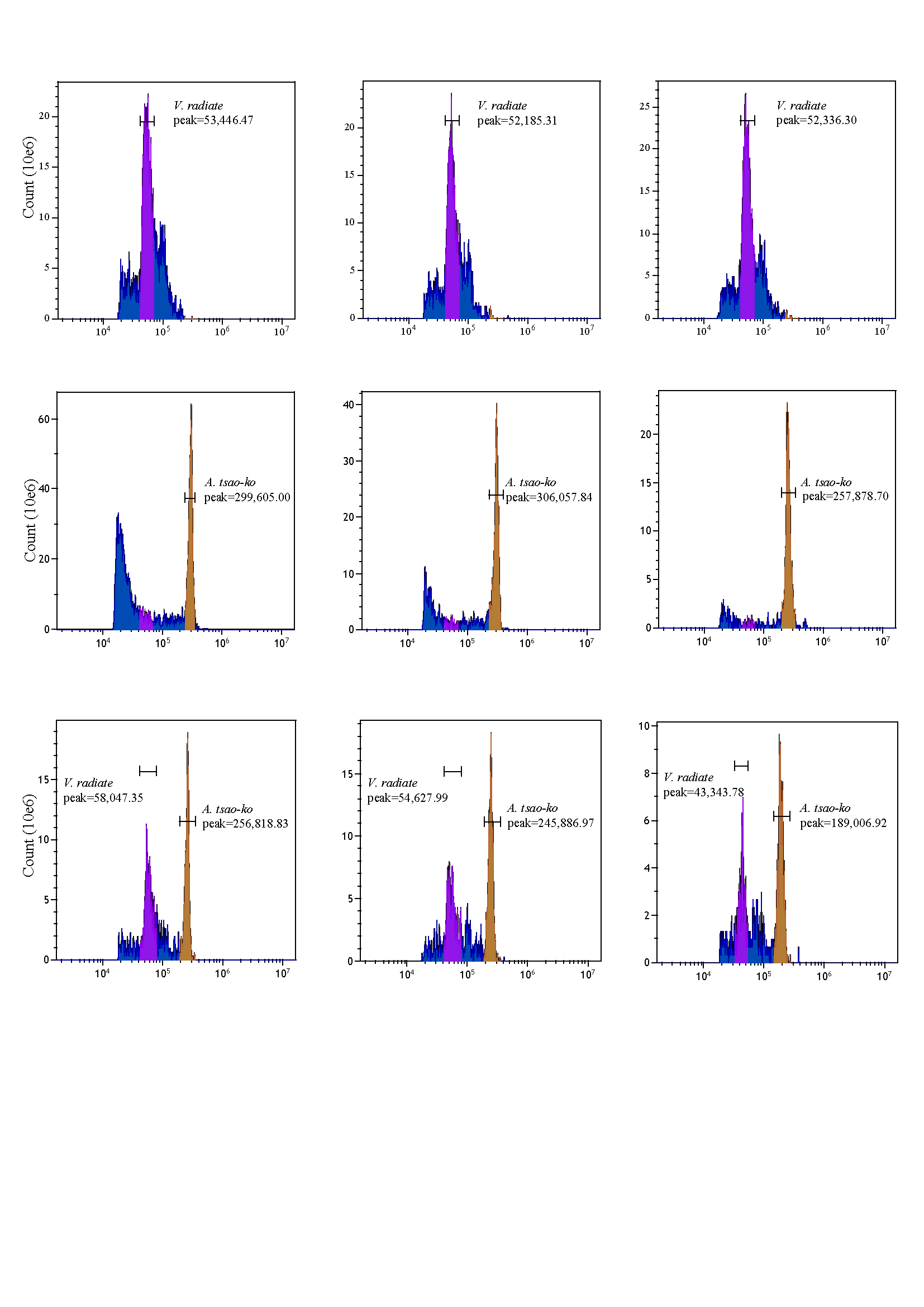


**Supplementary Figure 1.** Flow cytometry estimate of the *A. tsao-ko* genome size compared to reference standard of mung bean (*Vigna radiate*, 579 Mb). The average nuclei peak of mung bean is 52,656.03, and 287,847.18 in *A. tsao-ko*.


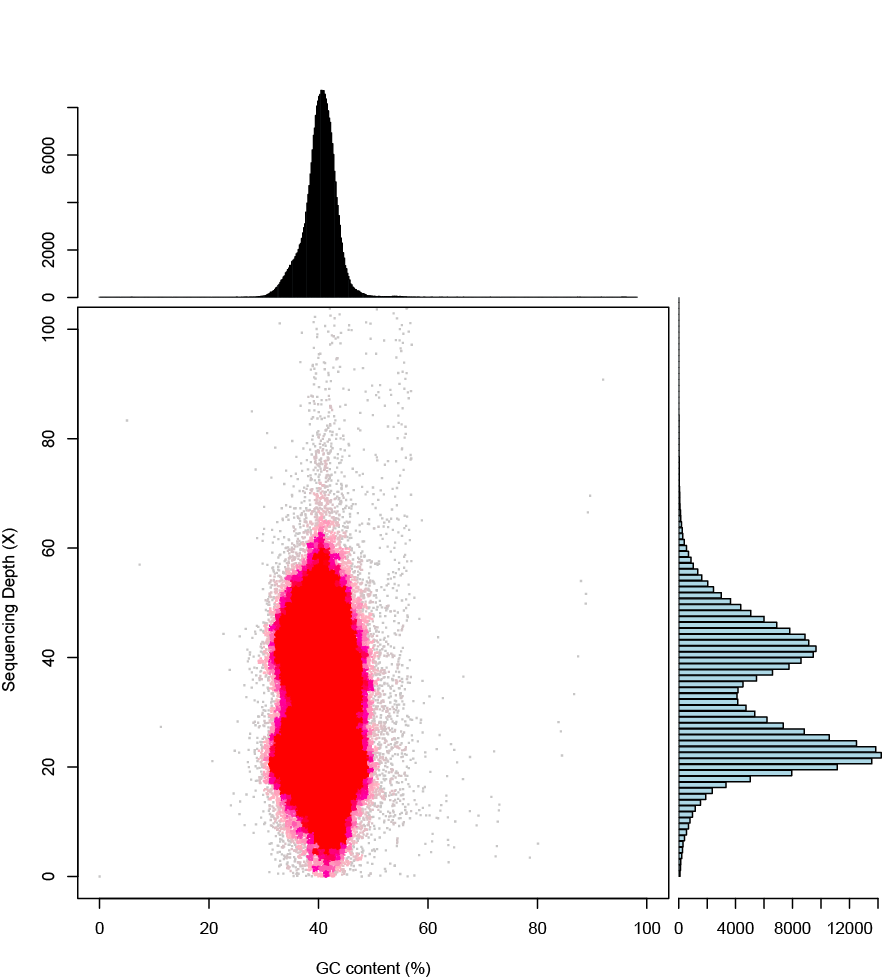


**Supplementary Figure 2**. Supplementary Figure 2. GC-depth distribution of *A. tsao-ko* genome. The x-axis represents the GC contents, and the y-axis represents the average sequencing depths. The x-marginal histogram on the top describes the distribution of GC contents, and the y-marginal histogram on the right describes the distribution of the sequencing depth.

**Supplementary Figure 3.** Schematic diagram of GO annotation of predicted genes.


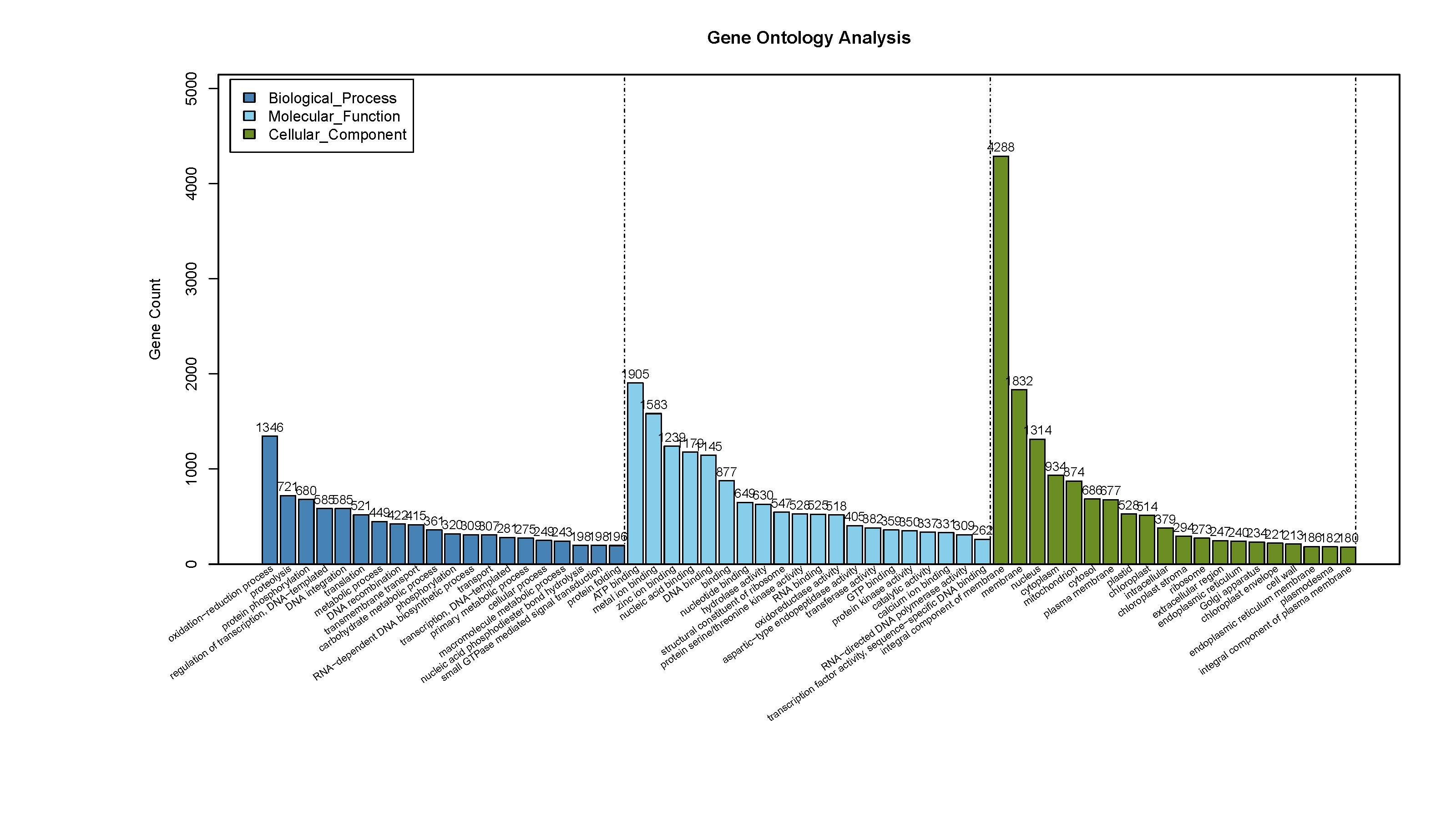


**
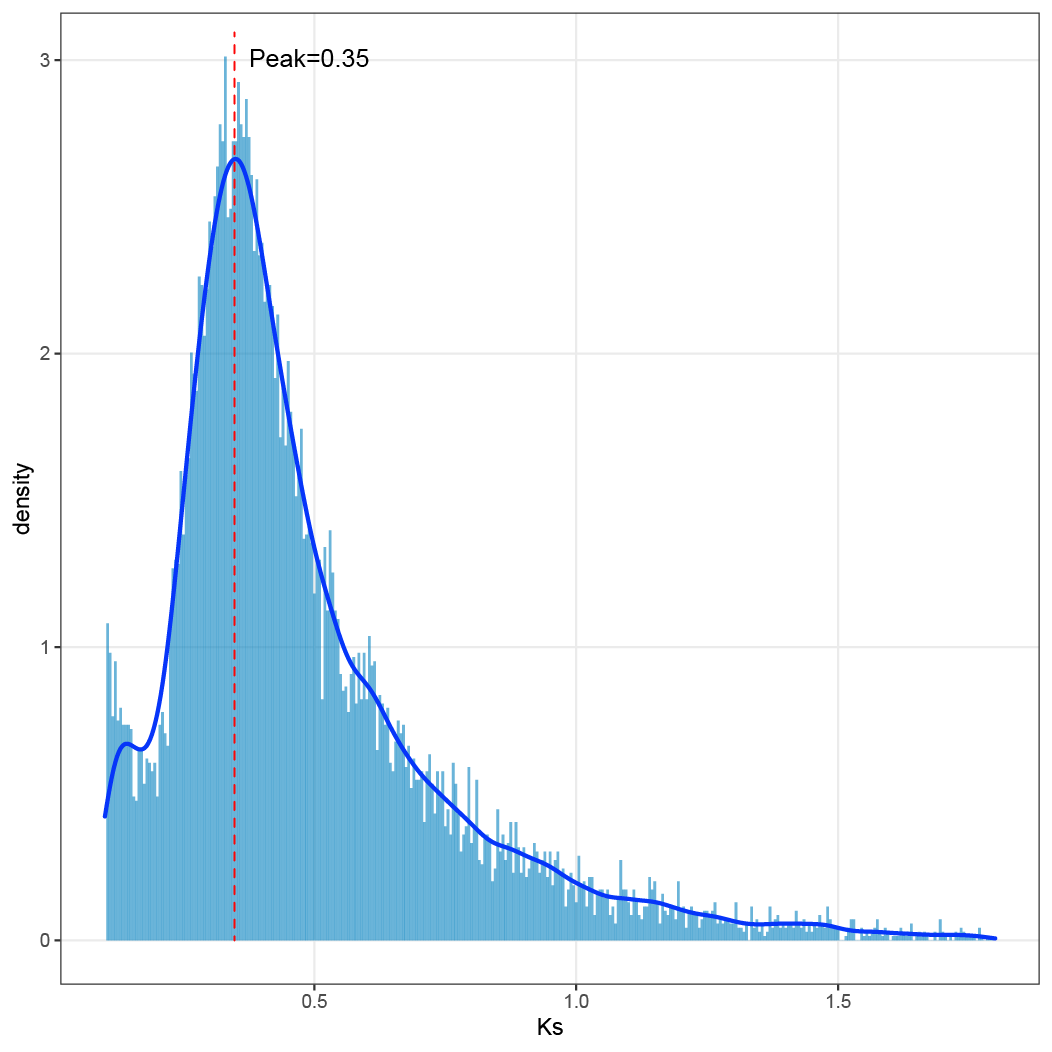
**

**Supplementary Figure 4.** Ks distribution of all the collinear genes in *A. tsao-ko* genome

**Supplementary Table 1: short paired-end reads generated in this study.**

| **Library** | **Library type** | **Raw reads** | **Raw bases** | **Clean reads** | **Clean bases** | **Read length** | **Raw Q30** |
| --- | --- | --- | --- | --- | --- | --- | --- |
| 20D11549DP3F401201PC | Survey-PCR-free | 181,084,257 | 54,325,277,100 | 181,047,713 | 54,271,776,276 | 150 | 93.84;86.30 |
| 20D11549DP3F401201PC | Survey-PCR-free | 30,380,412 | 9,114,123,600 | 30,375,925 | 9,105,584,726 | 150 | 91.64;82.70 |
| 20D11549DP3F401201PC | Survey-PCR-free | 140,342,592 | 42,102,777,600 | 140,321,758 | 42,068,406,372 | 150 | 92.67;86.84 |
| R14770Q2B365X65PA | TruseqmRNA-seq(Eu) | 25,068,458 | 7,520,537,400 | 24,910,068 | 7,421,497,968 | 150 | 94.43;91.40 |

Notes: One library was constructed for genome survey and was sequenced three times to ensure adequate data were obtained. R14770Q2B365X65PA is a library for RNA-seq.

**Supplementary Table 2: PacBio long reads generated in this study.**

| **Library** | **r64064_200910_001** | **r64066_200915_001** |
| --- | --- | --- |
| Polymerase Read Bases | 172,180,830,511 | 156,967,901,849 |
| Polymerase Reads | 6,549,023 | 5,862,790 |
| Unique Molecular Yield | 151,251,632,128 | 137,465,659,392 |
| Polymerase Read Length (mean) | 26,291 | 26,773 |
| Polymerase Read N50 | 41,461 | 41,873 |
| Subread Length (mean) | 22,362 | 22,751 |
| Subread N50 | 34,650 | 35,017 |
| Insert Length (mean) | 23,106 | 23,457 |
| Insert N50 | 35,712 | 36,028 |
| Number of of Control Reads | 3,928 | 9,348 |
| Control Read Length Mean | 39,607 | 33,144 |
| Control Read Concordance Mean | 0.863 | 0.864 |

Notes: Two library were prepared for PacBio long reads sequencing.

**Supplementary Table 3: Repetitive sequence characters in *A. tsao-ko genome***

| **Repeat type** | | **Classification** | **Number** | **Masked(bp)** | **Masked(%)** |
| --- | --- | --- | --- | --- | --- |
| Class I: Retrotransposon | LTR Retrotransposon | Copia | 1,128,494 | 961,225,168 | 35.55% |
|  |  | Gypsy | 589,786 | 518,096,310 | 19.16% |
|  |  | Others | 1,183,027 | 583,403,284 | 21.57% |
|  | Non-LTR Retrotransposon | LINE | 41,016 | 24,589,347 | 0.91% |
| Class II: DNA Transposon | Subclass I | DNA | 214,262 | 52,150,504 | 1.93% |
|  |  | DNA/CMC EnSpm | 13,754 | 13,172,366 | 0.49% |
|  |  | DNA/MULE MuDR | 14,471 | 11,846,830 | 0.44% |
|  |  | DNA/PIF Harbinger | 3,021 | 1,610,469 | 0.06% |
|  |  | DNA/hAT-Ac | 11,887 | 5,744,480 | 0.21% |
|  |  | DNA/hAT Tag1 | 3,766 | 2,060,965 | 0.08% |
|  |  | DNA/hAT Tip100 | 3,069 | 2,403,501 | 0.09% |
|  | Tandem Repeat | Simple repeat | 40,625 | 18,943,960 | 0.70% |
|  | Unknown | - | 880,752 | 215,552,579 | 7.97% |
|  | Other | - | 49 | 6,971 | 0.00% |
|  | Total | - | 4,127,979 | 2,410,806,734 | 89.15% |

**Supplementary Table 4: Statistics of predicted genes in *A. tsao-ko genome***

| Number of genes | 54379 |
| --- | --- |
| Total genic length | 305,254,953 |
| Mean gene length | 5,613 |
| Number of transcripts | 57658 |
| Transcripts per gene | 1.1 |
| Total transcript length | 85,726,499 |
| Mean transcript length | 1486 |
| Number of exons | 308,435 |
| Exons per transcript | 5.3 |
| Mean exon length | 277 |
| Number of coding exons | 300,939 |
| Number of introns | 250,777 |
| Mean intron length | 993 |
| Total cds length | 78,325,138 |
| Mean CDS length | 1,358 |

**Supplementary Table 5: Statistics of non-coding RNA in *A. tsao-ko genome***

| **Type** | **number** |
| --- | --- |
| Cis-reg::riboswitch | 3 |
| ribozyme | 1 |
| sRNA | 12 |
| Gene | 63 |
| snRNA::snoRNA::CD-box | 6,138 |
| snRNA::splicing | 133 |
| rRNA | 3,335 |
| Cis-reg | 19 |
| antisense | 15 |
| snRNA::snoRNA::HACA-box | 78 |
| tRNA | 1,417 |
| Intron | 112 |
| miRNA | 234 |

**Supplementary table S6: Statistics of gene cluster analysis**

| Species | Acom | Aoff | Atha | Atri | Atso | Macu | Mbal | Osat | Sbic |
| --- | --- | --- | --- | --- | --- | --- | --- | --- | --- |
| Number of genes | 22,166 | 26,460 | 27,444 | 17,022 | 54,379 | 30,737 | 33,021 | 28,577 | 28,120 |
| Number of genes in orthogroups | 20,205 | 22,598 | 21,346 | 15,528 | 42,309 | 29,694 | 25,779 | 25,043 | 24,726 |
| Number of unassigned genes | 1,961 | 3,862 | 6,098 | 1,494 | 12,070 | 1,043 | 7,242 | 3,534 | 3,394 |
| Percentage of genes in orthogroups | 91.2 | 85.4 | 77.8 | 91.2 | 77.8 | 96.6 | 78.1 | 87.6 | 87.9 |
| Percentage of unassigned genes | 8.8 | 14.6 | 22.2 | 8.8 | 22.2 | 3.4 | 21.9 | 12.4 | 12.1 |
| Number of orthogroups containing species | 12,310 | 11,667 | 11,568 | 11,658 | 12,342 | 13,468 | 12,892 | 13,935 | 13,856 |
| Percentage of orthogroups containing species | 72.8 | 69 | 68.4 | 68.9 | 73 | 79.6 | 76.2 | 82.4 | 81.9 |
| Number of species-specific orthogroups | 17 | 29 | 75 | 19 | 38 | 1 | 11 | 18 | 24 |
| Number of genes in species-specific orthogroups | 79 | 480 | 976 | 79 | 527 | 2 | 59 | 92 | 146 |
| Percentage of genes in species-specific orthogroups | 0.4 | 1.8 | 3.6 | 0.5 | 1 | 0 | 0.2 | 0.3 | 0.5 |

Notes: Acom: *Ananas comosus*; Aoff: *Asparagus officinalis*; Atha: *Arabidopsis thaliana*; Atri: *Amborella trichopoda*; Atso: *Amomum tsaoko*; Macu: *Musa acuminate*; Mbal: *Musa balbisiana*; Osat: *Oryza sativa*; Sbic: *Sorghum bicolor*

**Supplementary table S7: Go enrichment analysis of *A. tsao-ko* expanded genes**

| ID | Description | *P*-adjust |
| --- | --- | --- |
| GO:0015074 | DNA integration | 5.28E-97 |
| GO:0006310 | DNA recombination | 7.38E-93 |
| GO:0005488 | binding | 1.68E-87 |
| GO:0044260 | cellular macromolecule metabolic process | 1.86E-53 |
| GO:0004523 | RNA-DNA hybrid ribonuclease activity | 2.16E-49 |
| GO:0004519 | endonuclease activity | 3.1E-48 |
| GO:0016779 | nucleotidyltransferase activity | 9.79E-47 |
| GO:0008233 | peptidase activity | 2.18E-45 |
| GO:0090502 | RNA phosphodiester bond hydrolysis, endonucleolytic | 1.92E-43 |
| GO:0046718 | viral entry into host cell | 2.76E-42 |
| GO:0090305 | nucleic acid phosphodiester bond hydrolysis | 3.47E-39 |
| GO:0090304 | nucleic acid metabolic process | 6.08E-39 |
| GO:0004518 | nuclease activity | 7.82E-37 |
| GO:0043531 | ADP binding | 4.33E-28 |
| GO:0032550 | purine ribonucleoside binding | 3.77E-22 |
| GO:0032559 | adenyl ribonucleotide binding | 3.77E-22 |
| GO:0006259 | DNA metabolic process | 2.78E-21 |
| GO:0043168 | anion binding | 4.48E-21 |
| GO:0003964 | RNA-directed DNA polymerase activity | 9.65E-17 |
| GO:0006278 | RNA-dependent DNA biosynthetic process | 2.87E-16 |
| GO:0016740 | transferase activity | 2.19E-14 |
| GO:0000786 | nucleosome | 7.55E-11 |
| GO:0008553 | hydrogen-exporting ATPase activity, phosphorylative mechanism | 7.92E-11 |
| GO:0008661 | 1-deoxy-D-xylulose-5-phosphate synthase activity | 1.03E-10 |
| GO:0044550 | secondary metabolite biosynthetic process | 1.64E-10 |
| GO:0006754 | ATP biosynthetic process | 2.79E-10 |
| GO:0005992 | trehalose biosynthetic process | 3.41E-10 |
| GO:0004497 | monooxygenase activity | 6.3E-09 |
| GO:0010052 | guard cell differentiation | 8.51E-08 |
| GO:0016829 | lyase activity | 9.99E-08 |
| GO:0004478 | methionine adenosyltransferase activity | 1.09E-07 |
| GO:0006556 | S-adenosylmethionine biosynthetic process | 1.09E-07 |
| GO:0000790 | nuclear chromatin | 1.81E-07 |
| GO:0006342 | chromatin silencing | 1.81E-07 |
| GO:0005200 | structural constituent of cytoskeleton | 3.04E-07 |
| GO:0048027 | mRNA 5'-UTR binding | 5.79E-07 |
| GO:0003774 | motor activity | 1.18E-06 |
| GO:0016459 | myosin complex | 1.18E-06 |
| GO:0006730 | one-carbon metabolic process | 1.48E-06 |
| GO:0016131 | brassinosteroid metabolic process | 2.98E-06 |
| GO:0004190 | aspartic-type endopeptidase activity | 5.63E-06 |
| GO:0004805 | trehalose-phosphatase activity | 5.63E-06 |
| GO:0046982 | protein heterodimerization activity | 1.02E-05 |
| GO:0016004 | phospholipase activator activity | 1.63E-05 |
| GO:0070181 | small ribosomal subunit rRNA binding | 4.19E-05 |
| GO:0007017 | microtubule-based process | 5.6E-05 |
| GO:0006452 | translational frameshifting | 8.33E-05 |
| GO:0045901 | positive regulation of translational elongation | 8.33E-05 |
| GO:0045905 | positive regulation of translational termination | 8.33E-05 |
| GO:0009987 | cellular process | 0.000103 |
| GO:0015853 | adenine transport | 0.000107 |
| GO:0015854 | guanine transport | 0.000107 |
| GO:0030427 | site of polarized growth | 0.000107 |
| GO:0075133 | modulation by symbiont of host calcium or calmodulin-mediated signal transduction | 0.000107 |
| GO:0009812 | flavonoid metabolic process | 0.000125 |
| GO:0006351 | transcription, DNA-templated | 0.000185 |
| GO:0016709 | oxidoreductase activity, acting on paired donors, with incorporation or reduction of molecular oxygen, NAD(P)H as one donor, and incorporation of one atom of oxygen | 0.000185 |
| GO:0016787 | hydrolase activity | 0.000191 |
| GO:0019904 | protein domain specific binding | 0.0002 |
| GO:0010928 | regulation of auxin mediated signaling pathway | 0.000218 |
| GO:0019843 | rRNA binding | 0.000239 |
| GO:0016491 | oxidoreductase activity | 0.000239 |
| GO:0020037 | heme binding | 0.000274 |
| GO:0004170 | dUTP diphosphatase activity | 0.000313 |
| GO:0071949 | FAD binding | 0.000388 |
| GO:0004311 | farnesyltranstransferase activity | 0.000528 |
| GO:0035145 | exon-exon junction complex | 0.000528 |
| GO:0006636 | unsaturated fatty acid biosynthetic process | 0.000568 |
| GO:0019903 | protein phosphatase binding | 0.000568 |
| GO:1902456 | regulation of stomatal opening | 0.000568 |
| GO:0046916 | cellular transition metal ion homeostasis | 0.000568 |
| GO:0006887 | exocytosis | 0.000596 |
| GO:0044238 | primary metabolic process | 0.000887 |
| GO:0003825 | alpha, alpha-trehalose-phosphate synthase (UDP-forming) activity | 0.000887 |
| GO:1901601 | strigolactone biosynthetic process | 0.000887 |
| GO:0044267 | cellular protein metabolic process | 0.001124 |
| GO:0005874 | microtubule | 0.001177 |
| GO:0016705 | oxidoreductase activity, acting on paired donors, with incorporation or reduction of molecular oxygen | 0.001547 |
| GO:0005345 | purine nucleobase transmembrane transporter activity | 0.00155 |
| GO:1904823 | purine nucleobase transmembrane transport | 0.00155 |
| GO:0043170 | macromolecule metabolic process | 0.001607 |
| GO:0022857 | transmembrane transporter activity | 0.001669 |
| GO:0030976 | thiamine pyrophosphate binding | 0.001669 |
| GO:0000028 | ribosomal small subunit assembly | 0.001702 |
| GO:0006839 | mitochondrial transport | 0.001861 |
| GO:0010223 | secondary shoot formation | 0.001861 |
| GO:0007264 | small GTPase mediated signal transduction | 0.002303 |
| GO:2000377 | regulation of reactive oxygen species metabolic process | 0.002401 |
| GO:0006906 | vesicle fusion | 0.002431 |
| GO:0046914 | transition metal ion binding | 0.002641 |
| GO:0042744 | hydrogen peroxide catabolic process | 0.002669 |
| GO:0002221 | pattern recognition receptor signaling pathway | 0.002895 |
| GO:0004013 | adenosylhomocysteinase activity | 0.002895 |
| GO:0004363 | glutathione synthase activity | 0.002895 |
| GO:0015696 | ammonium transport | 0.002895 |
| GO:0043295 | glutathione binding | 0.002895 |
| GO:0000184 | nuclear-transcribed mRNA catabolic process, nonsense-mediated decay | 0.003331 |
| GO:0004869 | cysteine-type endopeptidase inhibitor activity | 0.003331 |
| GO:1902600 | hydrogen ion transmembrane transport | 0.003784 |
| GO:0016114 | terpenoid biosynthetic process | 0.003786 |
| GO:0032440 | 2-alkenal reductase [NAD(P)] activity | 0.006322 |
| GO:0046080 | dUTP metabolic process | 0.006322 |
| GO:0010359 | regulation of anion channel activity | 0.006322 |
| GO:0071704 | organic substance metabolic process | 0.006821 |
| GO:0019005 | SCF ubiquitin ligase complex | 0.006821 |
| GO:0065007 | biological regulation | 0.009347 |
| GO:0004022 | alcohol dehydrogenase (NAD) activity | 0.01025 |
| GO:0031966 | mitochondrial membrane | 0.01025 |
| GO:0047334 | diphosphate-fructose-6-phosphate 1-phosphotransferase activity | 0.01025 |
| GO:0000462 | maturation of SSU-rRNA from tricistronic rRNA transcript (SSU-rRNA, 5.8S rRNA, LSU-rRNA) | 0.010471 |
| GO:0003959 | NADPH dehydrogenase activity | 0.0106 |
| GO:0006226 | dUMP biosynthetic process | 0.0106 |
| GO:0006750 | glutathione biosynthetic process | 0.0106 |
| GO:0008274 | gamma-tubulin ring complex | 0.0106 |
| GO:0033566 | gamma-tubulin complex localization | 0.0106 |
| GO:0046081 | dUTP catabolic process | 0.0106 |
| GO:0005315 | inorganic phosphate transmembrane transporter activity | 0.011309 |
| GO:0033609 | oxalate metabolic process | 0.011309 |
| GO:0046564 | oxalate decarboxylase activity | 0.011309 |
| GO:0000381 | regulation of alternative mRNA splicing, via spliceosome | 0.011436 |
| GO:0052692 | raffinose alpha-galactosidase activity | 0.011436 |
| GO:0004427 | inorganic diphosphatase activity | 0.011661 |
| GO:0006796 | phosphate-containing compound metabolic process | 0.011661 |
| GO:0022627 | cytosolic small ribosomal subunit | 0.012142 |
| GO:0035556 | intracellular signal transduction | 0.012142 |
| GO:0016604 | nuclear body | 0.012142 |
| GO:0006508 | proteolysis | 0.01352 |
| GO:0004737 | pyruvate decarboxylase activity | 0.014845 |
| GO:0044422 | organelle part | 0.014845 |
| GO:0050737 | O-hydroxycinnamoyltransferase activity | 0.014845 |
| GO:0004601 | peroxidase activity | 0.015591 |
| GO:0035251 | UDP-glucosyltransferase activity | 0.015591 |
| GO:0006139 | nucleobase-containing compound metabolic process | 0.017311 |
| GO:0009958 | positive gravitropism | 0.018144 |
| GO:0006334 | nucleosome assembly | 0.020998 |
| GO:0006817 | phosphate ion transport | 0.021033 |
| GO:0043565 | sequence-specific DNA binding | 0.022489 |
| GO:0071805 | potassium ion transmembrane transport | 0.022537 |
| GO:0009816 | defense response to bacterium, incompatible interaction | 0.02333 |
| GO:0034641 | cellular nitrogen compound metabolic process | 0.02333 |
| GO:0031146 | SCF-dependent proteasomal ubiquitin-dependent protein catabolic process | 0.037958 |
| GO:0015079 | potassium ion transmembrane transporter activity | 0.040622 |
| GO:0005525 | GTP binding | 0.046464 |
| GO:0009911 | positive regulation of flower development | 0.047161 |
| GO:0051645 | Golgi localization | 0.047161 |
| GO:0090436 | leaf pavement cell development | 0.047161 |

**Supplementary table S8: KEGG enrichment analysis of *A. tsao-ko* expanded genes**

| Pathway ID | Description | *P*-adjust |
| --- | --- | --- |
| ko04626 | Plant-pathogen interaction | 1.57E-49 |
| ko00902 | Monoterpenoid biosynthesis | 1.30E-27 |
| ko04075 | Plant hormone signal transduction | 1.97E-21 |
| ko04740 | Olfactory transduction | 3.37E-10 |
| ko04745 | Phototransduction - fly | 3.37E-10 |
| ko04970 | Salivary secretion | 3.37E-10 |
| ko04971 | Gastric acid secretion | 3.37E-10 |
| ko00960 | Tropane, piperidine and pyridine alkaloid biosynthesis | 9.44E-10 |
| ko05130 | Pathogenic Escherichia coli infection | 5.96E-09 |
| ko04744 | Phototransduction | 2.17E-08 |
| ko00730 | Thiamine metabolism | 7.72E-08 |
| ko04670 | Leukocyte transendothelial migration | 7.84E-08 |
| ko04015 | Rap1 signaling pathway | 9.66E-08 |
| ko04722 | Neurotrophin signaling pathway | 9.66E-08 |
| ko04972 | Pancreatic secretion | 6.43E-07 |
| ko00980 | Metabolism of xenobiotics by cytochrome P450 | 1.27E-06 |
| ko00982 | Drug metabolism - cytochrome P450 | 2.49E-06 |
| ko00240 | Pyrimidine metabolism | 3.13E-06 |
| ko05204 | Chemical carcinogenesis | 2.87E-05 |
| ko04750 | Inflammatory mediator regulation of TRP channels | 5.74E-05 |
| ko04014 | Ras signaling pathway | 0.000119 |
| ko04713 | Circadian entrainment | 0.000637 |
| ko04540 | Gap junction | 0.000814 |
| ko05416 | Viral myocarditis | 0.001466 |
| ko05034 | Alcoholism | 0.001551 |
| ko04520 | Adherens junction | 0.002123 |
| ko00900 | Terpenoid backbone biosynthesis | 0.00322 |
| ko05100 | Bacterial invasion of epithelial cells | 0.004005 |
| ko05322 | Systemic lupus erythematosus | 0.004761 |
| ko00940 | Phenylpropanoid biosynthesis | 0.005924 |
| ko00908 | Zeatin biosynthesis | 0.005924 |
| ko05031 | Amphetamine addiction | 0.010954 |
| ko04664 | Fc epsilon RI signaling pathway | 0.014107 |
| ko00592 | alpha-Linolenic acid metabolism | 0.014188 |
| ko05214 | Glioma | 0.014672 |
| ko03320 | PPAR signaling pathway | 0.01515 |
| ko04145 | Phagosome | 0.025871 |
| ko04270 | Vascular smooth muscle contraction | 0.031817 |
| ko04916 | Melanogenesis | 0.036236 |
| ko00945 | Stilbenoid, diarylheptanoid and gingerol biosynthesis | 0.038069 |
| ko05014 | Amyotrophic lateral sclerosis (ALS) | 0.038069 |
| ko04912 | GnRH signaling pathway | 0.04534 |
| ko04062 | Chemokine signaling pathway | 0.046932 |
